# Assessing emergency department nurses' ability to communicate with angry patients and the factors that influence it

**DOI:** 10.3389/fpubh.2023.1098803

**Published:** 2023-01-26

**Authors:** Xi Chen, Yuting Zeng, Ling Jiang, Lingyun Tian, Jindong Yi, Haiyan He, Fang Li, Yanfang Long, Li Li

**Affiliations:** ^1^Teaching and Research Section of Clinical Nursing, Xiangya Hospital of Central South University, Changsha, Hunan, China; ^2^National Clinical Research Centre of Geriatric Disorders, Xiangya Hospital, Central South University, Changsha, Hunan, China; ^3^Department of Respiratory Medicine, National Key Clinical Specialty, Branch of National Clinical Research Center for Respiratory Disease, Xiangya Hospital, Central South University, Changsha, China; ^4^Department of Nursing, The First Affiliated Hospital of USTC, Division of Life Sciences and Medicine, University of Science and Technology of China, Hefei, Anhui, China

**Keywords:** emergency departments, patients, nurses, communication, anger, self efficacy

## Abstract

**Aims:**

To assess emergency department (ED) nurses' ability to communicate with angry patients and to explore the factors that influence nurses' communication skills.

**Design:**

A cross-sectional survey design.

**Methods:**

This study was conducted in November and December 2020. Stratified sampling was adopted to recruit ED nurses from 18 tertiary hospitals in western, eastern, and central China to complete an online questionnaire. The Nurses' Communication Ability with Angry Patients Scale (NCAAPS) and the General Self-Efficacy Scale were used to assess ED nurses' communication ability and self-efficacy, respectively. Descriptive statistics, the Mann–Whitney *U*-test, the Kruskal–Wallis H test, Spearman's correlation analysis, and the generalized linear model were used for data analysis.

**Results:**

A total of 679 valid questionnaires were collected. The mean total score for the NCAAPS was (3.79 ± 0.47), while the scores for its four dimensions were (3.87 ± 0.59) for communication skills, (3.82 ± 0.59) for anger perception, (3.79 ± 0.53) for self-preparation, (3.73 ± 0.54) for exploring the cause of anger. The generalized linear regression analysis result showed that a longer employment duration, previous communication ability training, and higher self-efficacy were significantly and independently associated with higher NCAAPS scores (*p* < 0.05).

**Conclusions:**

The mean total score and the four dimensions score for the NCAAPS were moderate. But there is still room for improvement in ED nurses' ability to communicate with angry patients. “Exploring the cause of anger” was the lowest score among the four dimensions. To improve ED nurses' ability to communicate with angry patients, future studies should focus on constructing specific communication training, improving nurses' ability to explore the cause of anger and self-efficacy.

**Impact:**

The findings of this study provide important insights into ED nurses' ability to communicate with angry patients and can thus guide the future development of intervention programmes to improve this ability among ED nurses.

## 1. Introduction

Workplace violence (WPV) in healthcare is among the most serious public health concerns worldwide given its increasing frequency and severity (1). The risk of WPV is highest in emergency departments (EDs) due to the characteristics of this work environment (e.g., long wait times, crowding, unrestricted movement of the public and understaffed EDs) (2). Nurses in particular, as frontline workers who are most commonly female, are at more risk of WPV than other healthcare professionals (3). Although the magnitude of WPV among ED nurses varies by country (3), the characteristics of WPV and ED nurses' responses to WPV are similar across countries and cultures with different sociocultural and economic status (4, 5). Studies have reported that 91.5% of ED nurses in Italy (6) and 87.4% of those in Oman (7) have experienced violence from various sources. The same problem occurs in China, where it was reported that the prevalence of WPV in ED nurses was 89.9% (8). WPV not only causes significant financial losses to hospitals in the form of medical bills, legal expenses, security costs, and missed labor work, but also poses tremendous emotional and relational costs to nurses, such as post-traumatic stress disorder, depression, fear, burnout, lack of job satisfaction, and intention to resign (1). Among multiple contributing factors that may lead to WPV against ED nurses, angry patient encounters and nurses' poor communication ability have been identified as essential factors that increase the risk of WPV.

Angry patient encounters are common in EDs as they are filled with anxious inpatients who require immediate medical care for urgent problems (9–11). Anger is one of the strongest predictors of aggressive behaviors (8, 12), and angry patients may have impaired judgement (10), which may increase the likelihood of out-of-control rage and violent outbursts, thus leading to serious WPV (13). Additionally, studies have documented that encounters with angry patients and their family members are a major contributor to nurses' stress and burnout, as they impose the extra burden of dealing with patients' and family members' angry outbursts in addition to providing medical care and treatment to the patients (5).

To prevent anger from escalating into WPV, it is important to help healthcare workers recognize patients' anger, ascertain the cause of the anger, and implement de-escalation techniques to prevent WPV (14, 15). Studies have shown that poor communication is an important trigger for anger in patients (14, 16). Importantly, most incidents of WPV are associated with outbursts of anger related to poor communication (13). Poor communication not only causes but also aggravates anger, which may in turn lead to WPV. The best strategy to deal with angry patients and prevent them from resorting to WPV is for the healthcare workers to implement effective communication skills (15). The World Health Organization has also highlighted that improving interpersonal and communication skills could prevent and defuse potential incidents of WPV (17).

EDs are full of angry and stressed people, and nurses must learn techniques for preventing this from escalating into violence. Given the protective role of ED nurses' good communication skills in WPV prevention in EDs, it is crucial to understand ED nurses' ability to communicate with angry patients and to identify the factors that contribute to this ability. There is a large body of research evaluating ED nurses' communication ability, and many intervention programmes have been designed to improve ED nurses' communication skills (18–20). However, most of these studies have focused on nurse–patient communications during difficult situations, such as when delivering bad news to patients and their family members (21) or communicating with patients with non-sedated mechanical ventilation (19) or intellectual disabilities (20). Few studies have reported on ED nurses' ability to communicate with angry patients, including their ability to identify and evaluate angry patients and to respond to angry patients (9, 14). Even fewer studies have focused on curriculum development to improve ED nurses' ability to deescalate patients' anger. Most of the studies on curriculum development have examined doctors in oncology and pediatrics departments (18, 22, 23). To the best of our knowledge, nursing schools in China generally lack a nurse–patient communication curriculum aiming at enhancing nurses' ability to communicate with angry patients.

The ability of communicating with an angry patient is reflected in many respects such as communication skills, anger perception, exploration of the cause of anger, self-preparation, and self-efficacy. Communication is influenced by many factors like listening strategies and ways of communication which can be divided into verbal communication and non-verbal communication (24). Previous studies show that communication skills are positively correlated with nurses' communication ability (25–27) and can be improved through training and practice (28). Anger perception refers to the ability to detecting and assessing patients' anger in the study (9). The first step for nurses to truly communicate with angry patients is to perceive anger. It is crucial for nurses to cope with patients' anger and aggression in an appropriate way (9). When a nurse perceives the patients' anger or even aggression, his/her emotional support from is triggered for he/she is supposed to provide emotional support to patients in communication (29). Thus, if nurses want to communicate well with angry patients, they need to have sharp anger perception ability and can provide emotional support to angry patients. Exploration of the cause of anger is an embodiment of active communication for only active communicators will try to find out why the patients are angry (9, 16, 30). To figure out what causes patients' anger can effectively help nurses grasp the key to the communication and decide how to communicate with these angry patients. It is reported that the ability of exploring the cause of anger is positively correlated with the communication ability of nurses (16). Nurses' self-preparation also plays an important role in nurse-patient communication, which includes listening, empathizing, and assessing and controlling the environment (16, 30, 31). Full self-preparation can build up nurses' confidence and help them gather essential communication background information for targeted communication. A randomized control trial conducted in 2015 reported that poor self-preparation is a barrier to nurse-patient communication (32). To have a better communication between nurses and angry patients, self-preparation should be seriously considered. Self-efficacy refers to the level of self-confidence in executing actions or attaining specific performance outcomes (33). Studies show that self-efficacy is positively correlated with nurses' communication ability (34, 35). It is essential to enhance clinicians' self-efficacy in dealing with and responding to angry patients because it is the most important way to improve their ability to communicate with angry patients (23).

In summary, most studies have treated ED nurses' communication ability as a one-dimensional concept and failed to explore its various dimensions. Furthermore, most studies were conducted in Western countries with established legislation and policies on WPV and established hospital protocols on effective communication with angry patients, and thus, much less is known about the situation in China where WPV against ED nurses is highly prevalent, yet policies on WPV and training for nurses' communication skills are under-developed.

Although angry patient encounters and WPV are common in EDs and are known to be associated with ED nurses' ability to communicate with angry patients, to the best of our knowledge, there are few studies of ED nurses' ability to communicate with angry patients in China. In light of these research gaps, this study was conducted in Chinese tertiary hospitals to assess ED nurses' ability to communicate with angry patients using a multidimensional validated questionnaire, namely the Nurses' Communication Ability with Angry Patients Scale (NCAAPS), developed in our previous study (16) and to explore the factors influencing this ability among the ED nurses. Our findings may provide guidance for designing intervention programmes to improve nurses' ability to communicate with angry patients, which would help to reduce patient anger in hospitals and consequently decrease the incidence of out-of-control rage and WPV in EDs.

## 2. The study

### 2.1. Aims

This study aimed to (a) assess ED nurses' ability to communicate with angry patients and (b) identify potential factors influencing this ability among ED nurses.

### 2.2. Design

This study was a cross-sectional Web-based survey conducted at 18 tertiary hospitals across western China (Chongqing, Sichuan, and Gansu), eastern China (Beijing, Jiangsu, and Shandong), and central China (Hunan, Jiangxi, and Henan).

### 2.3. Participants

ED nurses were recruited from November to December 2020 of 18 tertiary hospitals over China using stratified sampling. All the participants met the following inclusion criteria: (a) registered nurses, (b) had worked independently in the ED of the sampled hospital for at least 3 months, and (c) were engaged in clinical nursing work during the investigation. Nurses undergoing internship and training at the EDs were excluded.

### 2.4. Sample size

Using the Kendall sample estimation method, we determined that a sample size at least 20 times the number of variables would be required for our multivariate analysis (36). Given that our multivariate analysis used 36 variables (including 7 socio-demographic variables and 29 variables from the questionnaire), and that 5% of the questionnaires may be invalid, we estimated that a sample size of at least 756 was required. From the recruited ED nurses, 767 completed the survey, satisfying the minimal sample size requirement. Eighty-eight nurses did not complete the survey, giving a response rate of 88.5%.

### 2.5. Measures

*Socio-demographic information* was collected using a basic information sheet, which included gender, age (years), duration of employment (years), professional title, marital status, educational level, and previous training experience in communication ability.

*Nurses' ability to communicate with angry patients* was measured using the NCAAPS compiled by Chen Xi (16). The NCAAPS consists of 19 items under four dimensions: anger perception (3 items), communication skills (3 items), exploring the cause of anger (6 items), and self-preparation (7 items). Each item is rated on a 5-point Likert scale from 1 = “strongly disagree” to 5 = “strongly agree.” The total score ranges from 19 to 95, with a higher score indicating a better ability to communicate with angry patients.

*Nurses' self-efficacy* was assessed using the General Self-Efficacy Scale (GSES) compiled by Schwarzer (33). The GSES consists of 10 items. Each item is rated on a 4-point Likert scale from 1 = “strongly disagree” to 4 = “strongly agree.” The total score ranges from 10 to 40 points, with a higher score indicating higher self-efficacy.

### 2.6. Data collection

Data were collected through an online questionnaire using the Wen Juan Xing (a professional online questionnaire survey tool used in China). Stratified sampling was used to select ED nurses from 18 tertiary hospitals across nine provinces covering western, eastern, and central parts of China. In the first stage, nine provinces were randomly selected from each of the three geographical regions. In the second stage, 6 third-grade general hospitals were randomly selected from each of the chosen provinces. In the third stage, 43 nurses were selected from the emergency department in each sampled hospital.

After obtaining approval from the ED heads in each sampled hospital, we first approached the head nurse of each ED through WeChat (the most popular instant messaging and social media app in China), introduced the study purpose and procedure to them, and provided them with the link to the questionnaire (https://www.wjx.cn/jq/35142644.aspx). Next, the head nurse of each ED explained the study to all of their nurses during the nurses' meeting and provided instructions on completing the survey. All eligible ED nurses were invited to participate in the study and provided written informed consent on the first page of the questionnaire before participating in the study. Nurses who declined to participate in the study were not given access to the online questionnaire.

### 2.7. Ethical considerations

The study was approved by the Xiang Ya Nursing College Institutional Review Board [2019045] of Central South University. Permission was obtained from the sample hospitals' top-level managers for data collection. All participants provided written informed consent for participation in the study, and the NCAAPS questionnaires were completed anonymously.

### 2.8. Statistical analyses

SPSS software (version 25.0; SPSS Inc.) was used to conduct the statistical analyses. The means with standard deviations (SDs) were calculated for the continuous variables, and frequencies with percentages were calculated for the categorical variables. Due to the skewness of the outcome variables, the descriptive statistics were calculated as medians and interquartile ranges (IQRs). Because the data collected did not exactly follow multivariate normal distributions, the Mann–Whitney *U*-test and Kruskal–Wallis *H*-test were conducted to compare non-parametric data distributions across groups, and Spearman's correlation coefficients were calculated to determine the relationship between the NCAAPS and GSES scores among ED nurses. Two-tailed *p* < 0.05 were considered statistically significant. Finally, the generalized linear model was used to further explore the factors influencing the NCAAPS scores of the ED nurses.

### 2.9. Validity, reliability, and rigor

To increase the validity of the questionnaire, IP address restriction technology was adopted to ensure that users with the same IP address could only complete the questionnaire once. Two research assistants were responsible for downloading and checking the questionnaire data from the Wen Juan Xing. During data cleaning (There is obvious information error of the questionnaire), 37 responses were excluded. A response time of < 4 min was considered invalid because a pilot test showed that a minimum of 4 min were required to complete the survey; on this basis, a further 24 responses completed in < 4 min were excluded. Additionally, a quality control question was set at the end of the questionnaire to screen for invalid responses. The participants were asked to make a self-assessment of their answers after completing the questionnaire, and questionnaires with the following responses were excluded from the final analysis: “For various reasons, I failed to fill in this questionnaire seriously and recommend excluding this questionnaire” and “For various reasons, I was distracted and can only ensure that a small part of the questionnaire was carefully completed.” On this basis, a further 27 responses were excluded). Because all items of this web base survey were settled must be filled if participants want to submit the questionnaire at the end. Thus, there is no missing data in this survey.

To ensure data accuracy, we used standard assessment tools that were well-validated in both previous studies and the present study. The original NCAAPS has shown good reliability with a Cronbach's alpha coefficient of 0.96 (0.81–0.89 for its four subscales) and a test–retest reliability coefficient of 0.74 in a sample of 456 ED nurses in China (16). In this study, the NCAAPS demonstrated good internal consistency with a Cronbach's alpha coefficient of 0.93 for the total scale and 0.77–0.88 for its four subscales. The Chinese version of the GSES has shown good reliability with a Cronbach's alpha of 0.95 and a test–retest reliability coefficient of 0.89 in a sample of 335 ED nurses in Taiwan (37). In this study, the GSES demonstrated good internal consistency with a Cronbach's alpha coefficient of 0.91.

## 3. Results

### 3.1. Characteristics of the respondents

**Figure 1:**
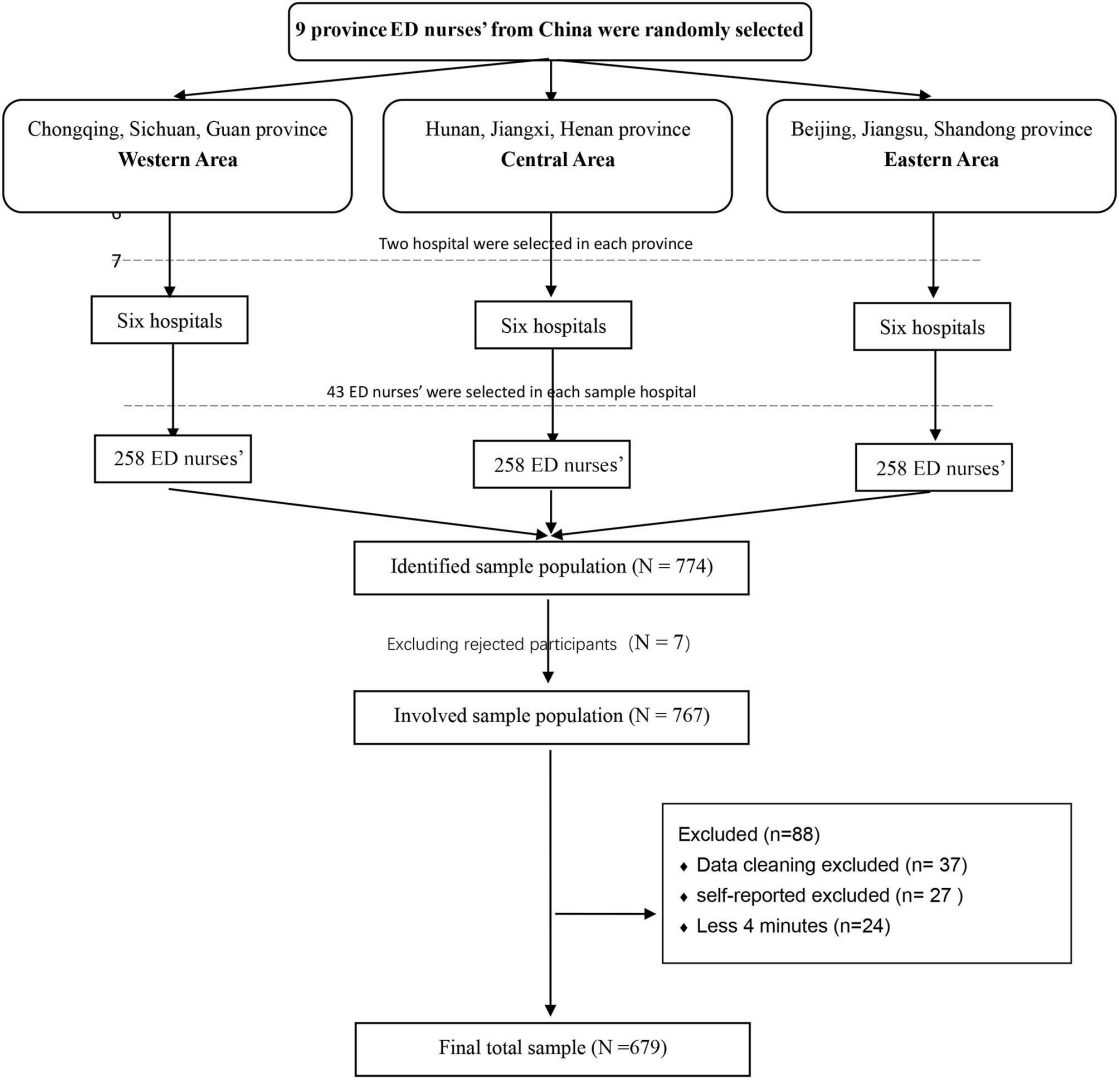
Flow diagram for the number of participants in each stage.

The characteristics of the respondents are shown in Table 1. A total of 767 questionnaires were collected, of which 679 were included in the data analysis, giving an effective response rate of 88.5%. Among the respondents, 81.7% were women. A detail of the participants' number included and excluded in each stage is shown in Figure 1. The respondents' age ranged from 21 to 52 years, with a median of 28 (IQR, 25–31) years. Most ED nurses had worked for < 5 years (59.9%), with a median duration of employment of 5 (IQR, 3–8) years. Nearly half of the respondents were senior nurses (48.9%) and married (44.6%), and most had an education level of bachelor's degree or above (77.2%). Most of the respondents (84.4%) had previous training in communication ability. The mean self-efficacy scores as measured by the GSES was 2.43 (SD = 0.55).

**Table 1:** Characteristics of ED nurses (*N* = 679).

**Characteristics**	**Groups**	***n* (%)/M(SD)**
Gender	Female	555 (81.7)
	Male	124 (18.3)
Age(years)	≤ 25	207 (30.5)
	26–35	402 (59.2)
	≥36	70 (10.3)
Length of employment (years)	≤ 5	407 (59.9)
	6–10	161 (23.7)
	≥11	111 (16.4)
Professional title	Primary nurse	228 (33.6)
	Senior nurse	332 (48.9)
	Nurse supervisor or above	119 (17.5)
Marital status	Married	303 (44.6)
	Single/divorced/widowed	376 (55.4)
Educational level	Associate diploma	155 (22.8)
	Bachelor's degree or above	524 (77.2)
Previous training experience in communication ability	Yes	573 (84.4)
	No	106 (15.6)
Self-efficacy (GSES)		2.43 (0.55)

### 3.2. NCAAPS scores

The mean total score for the NCAAPS was (3.79 ± 0.47). This was a moderately high score under a 5-level score system. The mean scores for its four individual dimensions were as follows: (3.87 ± 0.59) for communication skills, (3.82 ± 0.59) for anger perception, (3.79 ± 0.53) for self-preparation, and (3.73 ± 0.54) for exploring the cause of anger. Among the four dimensions, from the highest to the lowest ranking, “communication skills,” “anger perception,” “self-preparation,” and “exploring the cause of anger.” The score of the four dimensions were also moderately high. Table 2 lists the mean and median scores of the 19 items of the NCAAPS in the descending order of mean scores. There exists a wide range between the highest score item and the lowest score item, with almost 0.5 point range between them. The three items with the highest mean scores were item 19 “For unresolved patient anger, I can seek help from other medical team members in time” (seek help) (4.03 ± 0.70), item 1 “I can detect the anger of patients in time” (detect anger) (3.96 ± 0.74), and item 10 “I can use respectful words when communicating with angry patients” (use respectful words) (3.95 ± 0.67). The three items with the lowest mean scores were item 8 “I can alleviate the anger of patients” (alleviate anger) (3.56 ± 0.66), item 4 “I can actively communicate with the family members of angry patients or other contacts” (communicate actively) (3.65 ± 0.75), and item 17 “I can provide a calm emotional environment for angry patients in a timely manner” (provide a calm emotional environment) (3.67 ± 0.71).

**Table 2:** Scores of NCAAPS items (*N* = 679).

**Item number**	**Item content**	**Mean** **(SD)**	**M (*P*25, *P*75)**
19	For unresolved patient anger, I can seek help from other medical team members in time	4.03 (0.70)	4.00 (4.00, 4.00)
1	I can detect the anger of the patient in time	3.96 (0.74)	4.00 (4.00, 4.00)
10	I can use respectful words when communicating with angry patients	3.95 (0.67)	4.00 (4.00, 4.00)
11	I can use expressions and body language that demonstrate respect for angry patients	3.87 (0.69)	4.00 (4.00, 4.00)
6	I can find out from the patient's family or other contacts the cause of the patient's anger in time	3.85 (0.67)	4.00 (4.00, 4.00)
16	I can give rational feedback to the questions raised by angry patients	3.83 (0.66)	4.00 (3.00, 4.00)
2	I can identify potential factors that may cause anger in patients early	3.81 (0.69)	4.00 (3.00, 4.00)
5	I can find out from the patient the cause of their anger in time	3.81 (0.66)	4.00 (3.00, 4.00)
12	I can encourage angry patients to express themselves	3.80 (0.69)	4.00 (3.00, 4.00)
18	I can actively cooperate with family members or other contacts of angry patients to negotiate and resolve the anger of the patient	3.79 (0.68)	4.00 (3.00, 4.00)
7	I can understand the patient's past and needs in time	3.77 (0.68)	4.00 (3.00, 4.00)
14	I can listen carefully to patients without judgment	3.75 (0.78)	4.00 (3.00, 4.00)
15	When an angry patient expresses themselves unclearly, I can use feedback or repetition to clarify the patient's thoughts	3.75 (0.67)	4.00 (3.00, 4.00)
9	I can guide patients to express their demands rationally	3.71 (0.65)	4.00 (3.00, 4.00)
13	I can keep myself calm	3.69 (0.77)	4.00 (3.00, 4.00)
3	I can accurately assess the degree of patient anger	3.69 (0.70)	4.00 (3.00, 4.00)
17	I can provide a calm emotional environment for angry patients in a timely manner	3.67 (0.71)	4.00 (3.00, 4.00)
4	I can actively communicate with family members of angry patients or other contacts	3.65 (0.75)	4.00 (3.00, 4.00)
8	I can alleviate the anger of the patient	3.56 (0.66)	4.00 (3.00, 4.00)

M, median; P25, lower quartile; P75, upper quartil.

### 3.3. Univariate analyses of factors related to NCAAPS scores

Table 3 shows group comparisons of NCAAPS scores by sample characteristics. Significant differences in NCAAPS scores were found among participants with different ages, durations of employment, professional titles, and previous training in communication ability. In particular, the participants who were older, had longer durations of employment, had higher professional titles, and had previous training experience in communication ability had higher NCAAPS scores than their counterparts. Further, Spearman's correlation analysis showed that the GSES score was positively associated with the NCAAPS total score (r = 0.49, *p* < 0.01) and its four subscale scores (r_s_ = 0.37–0.44, *p* < 0.01).

**Table 3:** Differences in NCAAPS by sample characteristic (*N* = 679).

**Variables**	**Group**	**NCAAPS** **M (P25, P75)**	**Z/H**	***P***
Gender	Female	73.50 (67.00, 76.00)	−0.19	0.84^a^
	Male	73.00 (66.00, 76.00)		
Age (years)	≤ 25	73.00 (65.00, 76.00)	26.64	< 0.01^b^
	26–35	72.00 (66.00, 76.00)		
	≥36	76.00 (72.75, 81.25)		
Length of working experience (years)	≤ 5	72.00 (65.00, 76.00)	20.59	< 0.01^b^
	6–10	72.00 (65.00, 76.00)		
	≥11	75.00 (71.00, 80.00)		
Professional title	Primary nurse	72.00 (65.00, 76.00)	13.29	< 0.01^b^
	Senior nurse	72.00 (65.00, 76.00)		
	Nurse supervisor or above	75.00 (70.00, 78.00)		
Marital status	Married	72.00 (65.00, 76.00)	−1.89	0.06^a^
	Single/divorced/widowed	73.00 (68.00, 76.00)		
Educational level	Associate diploma	72.00 (64.00, 76.00)	–.55	0.58^a^
	Bachelor's degree or above	73.00 (67.00, 76.00)		
Previous training experience in communication ability	Yes	73.00 (67.00, 76.00)	−2.37	0.02^a^
	No	71.00 (64.00, 76.00)		

M is the median; P25 is the lower quartile; P75 is the upper quartile.

a The Mann–Whitney U-test.

b ruskal–Wallis H-test.

SD, standard deviation; NCAAPS, Nurses' Communication Ability with Angry Patients Scale.

Table 4 shows the results of the generalized linear regression to explore independent factors influencing NCAAPS scores. Among the eight factors included in the model, the following three factors remained significant after controlling for all other factors: duration of employment, previous training in communication ability, and self-efficacy. A longer duration of employment (β = 0.02–0.04, *p* = 0.04), previous training in communication ability (β = 0.03, *p* = 0.02), and higher self-efficacy (β = 0.11, *p* < 0.01) were significantly and independently associated with higher NCAAPS scores.

**Table 4:** Generalized linear model for factors associated with ED nurses NCAAPS (*N* = 679).

**Variables**	**Group**	**β**	**SE**	**Wald χ^2^**	***P***	**95% CI**
Intercept		1.05	0.02	2042.22	< 0.01	1.00–1.09
Gender	Female	0.02	0.01	2.30	0.13	−0.01–0.04
	Male	0^a^				
Age (years)	≥36	0.02	0.03	0.05	0.46	−0.03–0.07
	26–35	−0.01	0.01	0.68	0.41	−0.04–0.02
	≤ 25	0^a^				
Length of employment (years)	≥11	0.04	0.02	4.43	0.04	0.00–0.08
	6–10	0.02	0.01	4.05	0.04	0.00–0.05
	≤ 5	0^a^				
Professional title	Nurse supervisor or above	−0.02	0.02	1.61	0.21	−0.06–0.01
	Senior nurse	−0.02	0.01	1.73	0.19	−0.04–0.01
	Primary nurse	0^a^				
Marital status	Single/divorced/widowed	0.00	0.01	0.02	0.88	−0.02–0.02
	Married	0^a^				
Educational level	Bachelor degree or above	0.00	0.01	0.04	0.84	−0.02–0.02
	Associate diploma	0^a^				
Communication ability training experience	No	0.03	0.01	5.32	0.02	0.00–0.05
	Yes	0^a^				
GSES		0.11	0.01	223.95	< 0.01	0.10–0.13

CI, confidence interval; GSES, general self-efficacy scale.

a Reference.

## 4. Discussion

To the best of our knowledge, this is the first study to investigate the ability of ED nurses in mainland China to communicate with angry patients and the factors that influence this ability. One of the main findings of this study was that the ED nurses' mean total score for the NCAAPS was 3.79 out of 5. Although this result shows that the ability of ED nurses to communicate with angry patients was moderate, there is still much room for improvement, especially on the lowest-scoring dimensions and items.

Among the four dimensions of communication ability assessed by the NCAAPS, “communication skills” had the highest score, and “exploring the cause of anger” had the lowest score. There are many studies of nurses' communication ability training. However, rare previous training focus on how to train nurses to explore the cause of anger among patients. Thus, future studies should develop specific interventions to develop nurses' communication ability in exploring the cause of patients' anger. Our study showed that among the NCAAPS items, ED nurses had the highest scores for the items “seek help,” “detect anger,” and “use respectful words” with regard to angry patient encounters. This indicates that the ED nurses were able to detect patients' anger in time and deal with it appropriately by using respectful words and seeking help when necessary. Notably, the item “seek help” had the highest score, implying that most ED nurses preferred to seek help in dealing with angry patients. This suggests that once patients' anger was detected, the nurses felt threatened and took necessary measures to avoid confrontations with the angry patients. These results support previous findings (9) that the more anger was detected, the more threat ED nurses felt, and the more likely they were to seek help (e.g., call security). This tendency to seek help may reflect ED nurses' lack of experience in dealing with angry patients themselves and their fear of patients' anger escalating into WPV (38). This situation is further aggravated by ED nurses' limited time in care provision and high-workload (10). As a result, ED nurses may resort to external help to manage their fear of stressful encounters with angry patients rather than explore the cause of anger and address it directly. Our findings indicate that it is necessary to reduce ED nurses' workload, improve their work environment, increase the availability of helpful resources (such as security personnel), and alleviate their fear of angry patients to help them better communicate with patients.

Further, among the NCAAPS items, ED nurses had the lowest scores in the items “alleviate anger,” “communicate actively,” and “provide a calm emotional environment,” indicating the nurses need help to improve in these areas. The lack of skills in these areas is the main obstacle to effective communication with angry patients. Angry patients are common in EDs, and unresolved anger in patients may provoke anger in nurses (9), which will in turn impair the nurses' clinical reasoning and decision-making ability (10). As a result, ED nurses may not be able to provide a calm emotional environment and establish active communication with the angry patients, leading to unresolved patient anger.

One implication of the findings is that more training and guidance in dealing with angry patients in emergency situations should be provided to ED nurses. Consistently, previous studies have shown that curricula for the development of fundamental communication and de-escalation skills may help pediatric residents to more skilfully navigate interactions with angry caregivers (18, 31). EDs may also implement such de-escalation training for ED nurses to help them communicate more effectively with angry patients. Given that it is difficult to de-escalate patients' anger in EDs, ED nurses do not always succeed in providing a calm emotional environment for angry patients in time; therefore, ED nurses also need a legitimate place to vent their emotions. To that end, ED nurses could engage in non-judgemental “debriefing activity” after an angry patient encounter, as this would contribute to a healthy work environment that is non-judgemental and supportive at all levels of staffing. ED nurses have to deal with the most serious patients in the most complex situations (39) and are overwhelmed by surging workloads, high patient volume, and potential incidents of WPV (40). As a result, ED nurses may lack the appropriate space to communicate with angry patients without being harmed. It is thus suggested that EDs should provide a safer physical environment, which may include enhanced security (e.g., establishing metal detectors at entrances), increased team visibility (e.g., short, perpendicular corridors that enable staff to easily see what is happening throughout the ED and offer help where needed; eliminating view-obstructing features; and installing bulletproof glass), and adequate privacy (e.g., enclosed emergency rooms) (40).

Our study showed that a longer duration of employment was associated with a better ability to communicate with angry patients, which was consistent with a previous study on differences in nurses' competence between three generational nurse cohorts (41). ED nurses with the longest durations of employment were better at communicating with angry patients and may play an important role in providing anger de-escalation training for other less experienced nurses. Nurses' core knowledge and competencies develop over time and increase with increasing work experience (41, 42). Nurses with longer durations of employment have more experience of interacting with various types of patients and consequently more experience of dealing with angry patients. However, the health system is challenged by frequent nurse turnover and severe nurse shortages, especially in EDs (41). It is suggested that attractive incentives be provided to retain experienced nurses in the EDs and that standard training be provided for younger ED nurses to improve their communication ability. For instance, the hospital can encourage experienced nurses to provide training in communication skills for younger nurses and interns.

The finding that ED nurses with previous training in communication skills performed better than untrained nurses in communicating with angry patients is in line with a systematic review showing that nurses' communication ability can be improved with specific training (43). Further, our study showed that ED nurses found it difficult to alleviate patients' anger and often adopted an avoidant approach to dealing with angry patients, which also highlights the need for more training in self-preparation and anger de-escalation. Multiple training models have been developed and proven to be effective in improving nurses' communication ability. For instance, the HEAT (Hear, Empathize, Apologize, Take Action) communication strategy is a 1.5-hour-long workshop designed to train nurses in handling angry patient encounters and has been proven to be effective (31). A nine-step 90-min de-escalation curriculum showed that smooth management, attending to comfort, and showing respect to angry patients were positively associated with improved anger de-escalation performance (18). Immersive virtual reality has also been widely used in nurse and patient education to improve communication skills (44) and has been shown to be effective in improving anger management (45, 46). Future studies are warranted to determine the appropriate curriculum content, method, and evaluation and the ideal timing of interventions aimed at improving ED nurses' ability to communicate with angry patients.

In our study, higher self-efficacy among ED nurses was found to be associated with a better ability to communicate with angry patients, which is consistent with previous studies showing that nurses with higher self-efficacy are better at dealing with aggressive patients (23). Self-efficacy is an important psychological trait in nurses and is positively correlated with nurses' core competencies, including communication ability and interpersonal relationships, which are important in dealing with angry patients (42). Furthermore, previous studies have also revealed that nurses' communication ability has a positive effect on their self-efficacy, indicating the bidirectional association between self-efficacy and communication ability (34, 35). Thus, improving ED nurses' self-efficacy may enhance their communication ability, which in turn will further enhance their self-efficacy (26, 47). This finding suggests that ED nurses' communication ability may benefit from educational and training programmes that include components on fostering self-efficacy.

## 5. Limitations and research

Several limitations of this study should be noted. First, the cross-sectional study design made it impossible to derive causal inferences between influencing factors and communication ability, which need to be investigated in future longitudinal studies. Second, the quantitative study design lacked an in-depth exploration of ED nurses' experience of and perspectives on communication with angry patients, which warrant further qualitative research. Third, all of the data were self-reported and are thus vulnerable to recall bias; therefore, future studies combining other objective assessment tools, such as a digital video recorders, may enhance the external validity of the findings. Last, this study only investigates ED nurses' communication ability with angry patients. Thus, the study findings would be difficult to generalize to other department nurses.

## 6. Implications for nursing management

There is much room for improvement in ED nurses' ability to communicate with angry patients. Comprehensive and targeted training programmes focusing on multiple dimensions of ED nurses' communication ability should be designed and provided for ED nurses to help them better deal with angry patients. As an added advantage, improving ED nurses' ability to communicate with angry patients may prevent nurses from resigning from hospitals due to stress and burnout, thus reducing the nurse turnover rates in and the human resource costs incurred by hospitals.

## 7. Conclusion

Our findings indicate that there is much room for improvement in ED nurses' ability to communicate with angry patients. ED nurses with longer durations of employment, previous training in communication ability, and higher self-efficacy are better at communicating with angry patients. Therefore, future interventions for improving ED nurses' communication ability may be best served by including educational and training components on communication skills and self-efficacy, especially for young ED nurses with limited work experience.

## Data availability statement

The raw data supporting the conclusions of this article will be made available by the authors, without undue reservation.

## Ethics statement

The studies involving human participants were reviewed and approved by Institutional Review Board [2019045] by Nursing College of Central South University. The patients/participants provided their written informed consent to participate in this study.

## Author contributions

XC, YZ, and LL contributed to the conception and design of the study. LJ, LT, HH, FL, JY, and YL organized the database. XC, YZ, and LJ performed the statistical analysis. XC and YZ wrote the first draft of the manuscript. LJ, LT, HH, FL, JY, YL, and LL wrote sections of the manuscript. All authors contributed to manuscript revision, read, and approved the submitted version.
